# Multidimensional perspectives of geo-epidemiology: from interdisciplinary learning and research to cost–benefit oriented decision-making

**DOI:** 10.3389/fpubh.2024.1492426

**Published:** 2024-12-30

**Authors:** S. D. Smith, E. M. Geraghty, A. L. Rivas, F. O. Fasina, M. Kosoy, L. Malania, A. L. Hoogesteijn, J. M. Fair

**Affiliations:** ^1^Geospatial Research Services, Ithaca, NY, United States; ^2^Esri, Redlands, CA, United States; ^3^Center for Global Health, Internal Medicine, School of Medicine, University of New Mexico, Albuquerque, NM, United States; ^4^Department of Veterinary Tropical Diseases, Faculty of Veterinary Science, University of Pretoria, Pretoria, South Africa; ^5^Food and Agriculture Organization (FAO), Rome, Italy; ^6^KB One Health LLC, Fort Collins, CO, United States; ^7^National Center for Disease Control and Public Health, Tbilisi, Georgia; ^8^Department of Human Ecology, CINVESTAV, Merida, Yucatan, Mexico; ^9^Biosecurity, Los Alamos National Laboratory, Los Alamos, NM, United States

**Keywords:** geo-epidemiology, multidimensional analysis, emergence, geography, epidemics

## Abstract

Research typically promotes two types of outcomes (inventions and discoveries), which induce a virtuous cycle: something suspected or desired (not previously demonstrated) may become known or feasible once a new tool or procedure is invented and, later, the use of this invention may discover new knowledge. Research also promotes the opposite sequence—from new knowledge to new inventions. This bidirectional process is observed in geo-referenced epidemiology—a field that relates to but may also differ from spatial epidemiology. Geo-epidemiology encompasses several theories and technologies that promote inter/transdisciplinary knowledge integration, education, and research in population health. Based on visual examples derived from geo-referenced studies on epidemics and epizootics, this report demonstrates that this field may extract more (geographically related) information than simple spatial analyses, which then supports more effective and/or less costly interventions. Actual (not simulated) bio-geo-temporal interactions (never captured before the emergence of technologies that analyze geo-referenced data, such as geographical information systems) can now address research questions that relate to several fields, such as Network Theory. Thus, a new opportunity arises before us, which exceeds research: it also demands knowledge integration across disciplines as well as novel educational programs which, to be biomedically and socially justified, should demonstrate cost-effectiveness. Grounded on many bio-temporal-georeferenced examples, this report reviews the literature that supports this hypothesis: novel educational programs that focus on geo-referenced epidemic data may help generate cost-effective policies that prevent or control disease dissemination.

## Introduction

*Geo-epidemiology* may be described as an inter-disciplinary field that, based on geo-referenced and bio-dynamic data, attempts to prevent disease dissemination. Hoping to clarify the similarities and/or differences between geo-epidemiology and other related fields (1), here the literature on visualizations associated with disease dispersal is reviewed. Such an exercise is meant to emphasize that geo-epidemiology may promote *earlier, cost–benefit oriented, geography-* and *time-specific epidemiologic interventions*.

This review also describes considerations associated with technological development and *education*—in particular, how to teach novel interdisciplinary and decision-making oriented programs. They refer to *knowledge validation* which, in turn, is associated with *knowledge integration* (2).

The driving motivation for this report is that disease dispersal affects everybody, everywhere. As illustrated by COVID-19, avian influenza, and cholera (among many diseases), unless prevented, epidemics and enzootics may seriously affect humans and non-humans (3–5).

### From inventions to knowledge creation

While research promotes technological development, the opposite is also observed. For example, the emergence of geographical information systems (GIS) has fostered public health (6). GIS tools have been integral to infectious disease surveillance, vaccination campaign planning, and optimizing responses to public health crises such as COVID-19. These efforts have laid the groundwork for integrating spatial and temporal analyses more effectively.

## Geo-epidemiology vs. spatial epidemiology

While closely associated, space and geography are not synonymous. Space seems to be the *larger* category while geography is just one sub-domain. Yet, geography tends to be the *richer* concept because its contents and contexts are not always found in non-geographic space.

While *geography* refers to the study of the Earth (an actual, not a hypothetical entity), *space* refers to the study of any (actual or hypothetical) surface, located anywhere. Because our planet is composed of many non-randomly distributed elements (e.g., rivers, mountains, cities, forests, farms, and roads traveled by human and non-human individuals), geo-epidemiology differs substantially from the study of space, which could be imagined as a static environment—where there are no seasons and is inhabited by a homogeneously distributed population (6). Hence, diseases may be better understood when *geo-referenced* and *temporal data* are analyzed.

Accordingly, geo-epidemiology informs on *relationships* involving populations, rivers, forests, lakes, mountains, roads and many other geo-referenced entities, which are *dynamic* and may exist *before* diseases occur. Because geographical variables are non-hypothetical, they can be measured directly and because bio-geographical relationships change over time, the analysis of disease dispersal requires *multidimensional analyses* (7, 8).

While any tool used in spatial analysis, in principle, can also be used in geo-epidemiology, the opposite is not necessarily possible: patterns detected when geographical data are analyzed may be absent in (or missed by) spatial models. While spatial analysis is prone to uni-disciplinary/specialized (*reductionist*) approaches (8), geo-epidemiology is inherently inter/transdisciplinary and non-reductionist (9).

This report emphasizes *infectious diseases* that disseminate temporally and geographically, i.e., *epidemics* and *epizootics*. Such diseases usually utilize *pre-existing connecting* structures. To prevent or control them, *cost/benefit*-oriented analyses are necessary. Given the apparent lack of academic programs on geo-epidemiology, new educational (inter/transdisciplinary) programs seem needed.

*Inter/transdisciplinary knowledge* relates to but also differs from multidisciplinary knowledge. That is so because multi-disciplinarity does not necessarily integrate knowledge generated in several disciplines. For instance, the work conducted by electricians, plumbers, and carpenters in the process of rebuilding a house does not require previous integration of their expertise: following pre-established instructions, they could just apply what they previously learned. In contrast, interdisciplinary projects require the production of a novel solution that fits a specific (and usually novel) problem (9). When a substantial amount of new knowledge needs to be created to solve a specific problem, the term *trans-disciplinarity* tends to be used (10).

Because inter/trans-disciplinary knowledge cannot be communicated with a language grounded on any specialized field, new languages and templates may be required. A language common to many fields and constituencies may be facilitated when potential users share the same interest, context or field of application. Participatory approaches may promote the creation of such languages (11, 12).

Consequently, a process that identifies invalid, obsolete, and/or fragmented knowledge may foster problem solving (13). Non-reductionist, data-driven analysis of visually explicit information (such as geo-referenced data on disease dispersal) may promote inter/trans-disciplinary knowledge integration and prevent invalid inferences (14, 15).

## The design of this study

This material describes both the way diseases are investigated bio-geo-temporally, and how inter−/transdisciplinary educational and research processes can be promoted. Three sections describe: (i) features and/or properties of geo-*epidemiology*; (ii) *decision-making and applications* (in particular, those based on cost/benefit-related considerations), and (iii) concepts associated with education and research methods—especially those grounded on *visual* data.

### Section I. Features and properties of geo-epidemiology

#### Pre-established, geographically explicit connectivity may inform earlier

The ability to measure unambiguous connecting structures that predate disease emergence is a feature that distinguishes geo-epidemiology from simple spatial approaches. While *connectors* are associated with *contacts*, they also differ from one another (16). While *contacts* are human or non-human *individuals*, *connectors* refer to *physical structures* individuals utilize while traveling and/or contacting one another, e.g., a tunnel, road, bridge, airplane, etc. Unlike approaches that focus on *contacts*, methods that describe *connectivity* do not need to identify individuals—identifying the *locations* of *connectors* (which *precede* the occurrence of infections) may suffice. While the contact-oriented approach depends on highly variable data (which may be available only after a crucial event has occurred), connectivity-oriented approaches only need to collect data on pre-existing connectors and, consequently, they provide information inherently prognostic.

Because geo-epidemiology reflects how diseases disseminate, disease dispersal is necessarily based on *pre-established connecting networks*. Roads, rivers, railroads are examples of pre-established connectors. Only by using connecting structures that predate the emergence (or re-emergence) of a pathogen, can a disease spread out.

**Figure 1:**
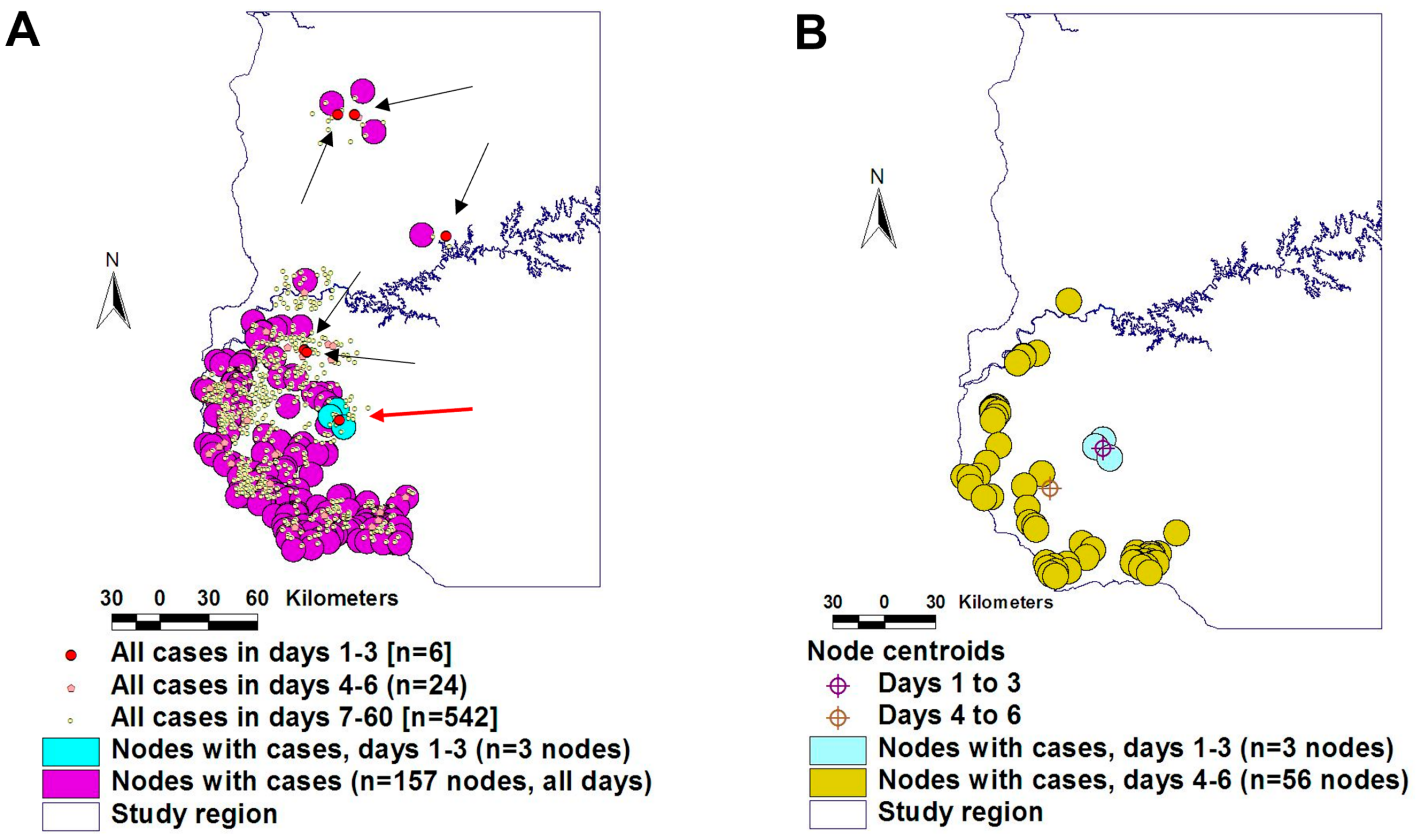
Geo-referenced and temporal evaluation of earlier theories. The assumption that the ‘index case’ (the first reported case) originates disease dissemination was not supported by the evidence: only one of the six cases reported in the first transmission cycle (estimated in 3 days) was located on a highway node (red dot inserted within a sky-blue circle, **A**). The data reported in the second transmission cycle (days 4–6) support the hypothesis that the later disease dissemination originated in the highly connected case **(B)**. Source: reference (16).

Therefore, some earlier concepts (such as the *time* and *location* of the first or ‘index’ case) are not necessarily valid because—when cases are reported in places where more than one connecting structures exist—, epidemic or enzootic processes tend to occur (16). Figure 1A illustrates this concept: by plotting actual data on disease dispersal in relation to connecting structures (e.g., the highway network), it is observed that the case regarded as the first (‘index’) case was not well connected–only one of the first 6 cases was located near to or on a highway intersection (a ‘node’ that facilitates two or more dissemination routes, Figure 1A). In contrast, the only case located on the connecting structure explained the subsequent disease dispersal: at days 4–6, the centroid of all epidemic nodes moved into a highly connected dissemination structure (Figure 1B). Thus, to explore disease dispersal not only *geographical* information is required (on the road network in this case) but also data on the estimated transmission cycle of the pathogen—up to 3 days, in this case.

#### Network theory-related properties

The distinction between pre-existing *connectivity* and *contacts* matters in decision-making. When interventions are designed, it may be difficult to identify the specific contact that could link a specific case with a susceptible individual. In contrast, the specific (geo-referenced) *connecting* structure (i.e., the ‘node’ that, if blocked, could prevent disease spread) may be easily identified. However, such an identification requires distinguishing ‘average’ from ‘highly connected’ epidemic node-related cases (16–18).

Hence, rapidly elucidating the most likely connecting link that promotes disease dispersal is critical for planning and delivering effective interventions. The underlying principle is that, when the connecting structure associated with a specific disease outbreak is presumptively identified, it is then possible to conceive targeted responses, which are likely to be more effective, more rapidly implemented and/or less costly than non-specific and/or static ones (16).

To take advantage of such a possibility, the study of Network Theory seems required. Applied to epidemiology, Network Theory can be described by several properties, including: (i) *Pareto’s 20:80 distribution*, (ii) *synchronicity,* and (iii) *directionality*. These properties have been empirically observed in three epidemic processes that affected bovine, avian, and human species, respectively (16, 19).

Pareto’s ‘20:80’ distribution refers to the fact that not all epidemic nodes equally influence disease dispersal: only a minority (~20%) of the earlier cases generates most (~80%) of the later cases (20). Consequently, not all epidemic nodes are epidemiologically identical. Because some epidemic nodes are more influential than others, they should be distinguished.

**Figure 2:**
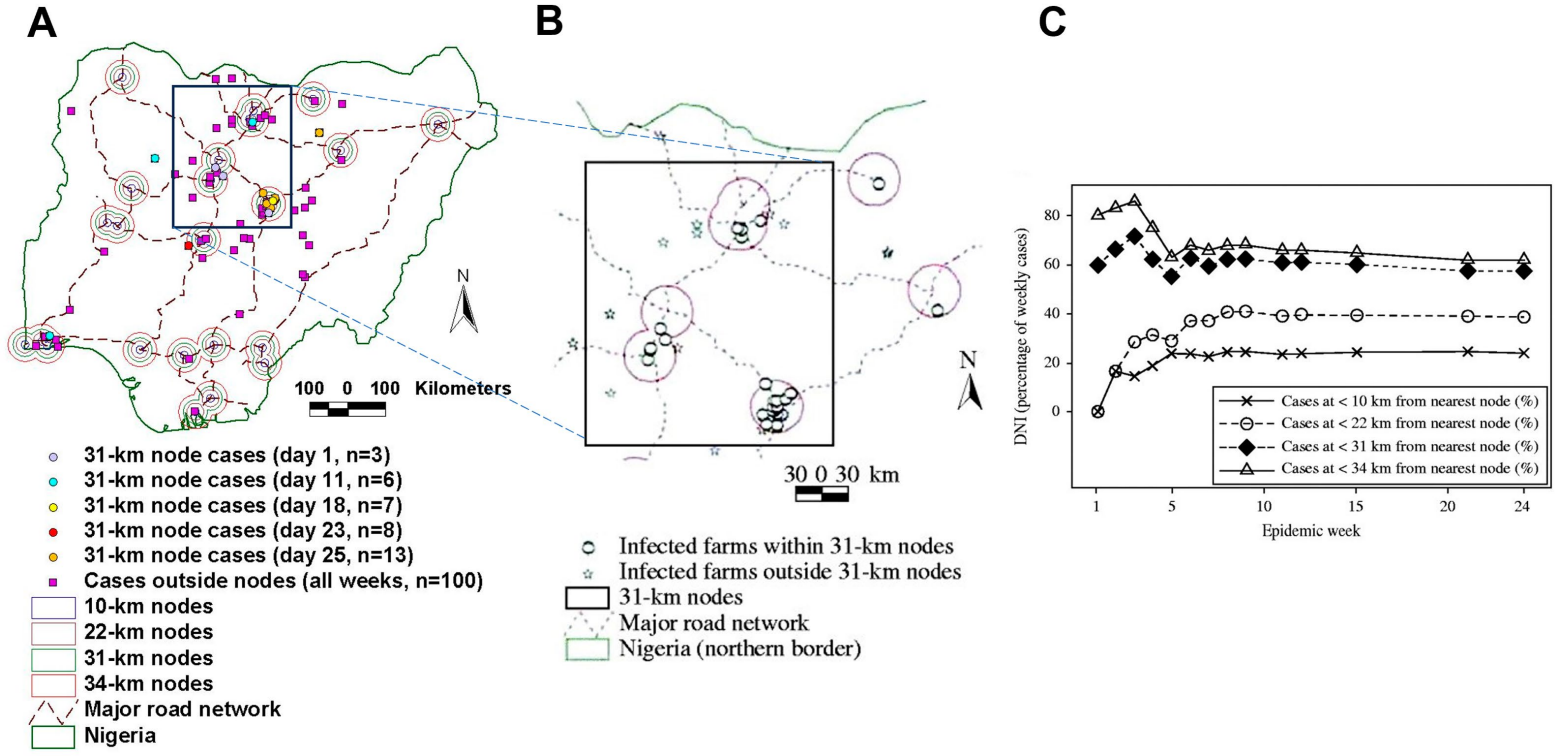
Geo-temporal detection of epidemic nodes. Avian Influenza (H5N1) epidemic nodes of the 2006 Nigerian epizootic were the smallest circles that, earlier and longer, contained more cases and were connected (they included a highway intersection, **A**). Throughout the epidemic, Avian Influenza (AI) epidemic nodes were 31-km radius circles centered on intersections of the national highway network **(B)**. Such nodes included ~60% of all AI cases **(C)**. Source: reference (16).

Time- and geography-specific differentiation of epidemic nodes can be objectively determined: they are the sites that include both a connecting structure (e.g., a highway intersection) and the highest percentage of cases at a specific bio-temporal (disease transmission-related) cycle. Operational definitions of what epidemic nodes are and how their influence can be distinguished can be made for a specific disease and environment (Figure 2).

For example, the analysis of geographical interactions—such as the relationships among road density, case density, relative length of roads per area unit—may identify ‘hubs’ or ‘nodes’ of relationships that, if identified before epidemics occur, could lead to anticipatory measures. Such approaches could lead to global anticipatory mapping of all such potential ‘facilitators’ of disease dispersal (21).

#### Data on geo-bio-temporal interactions may re-evaluate previous theories

**Figure 3:**
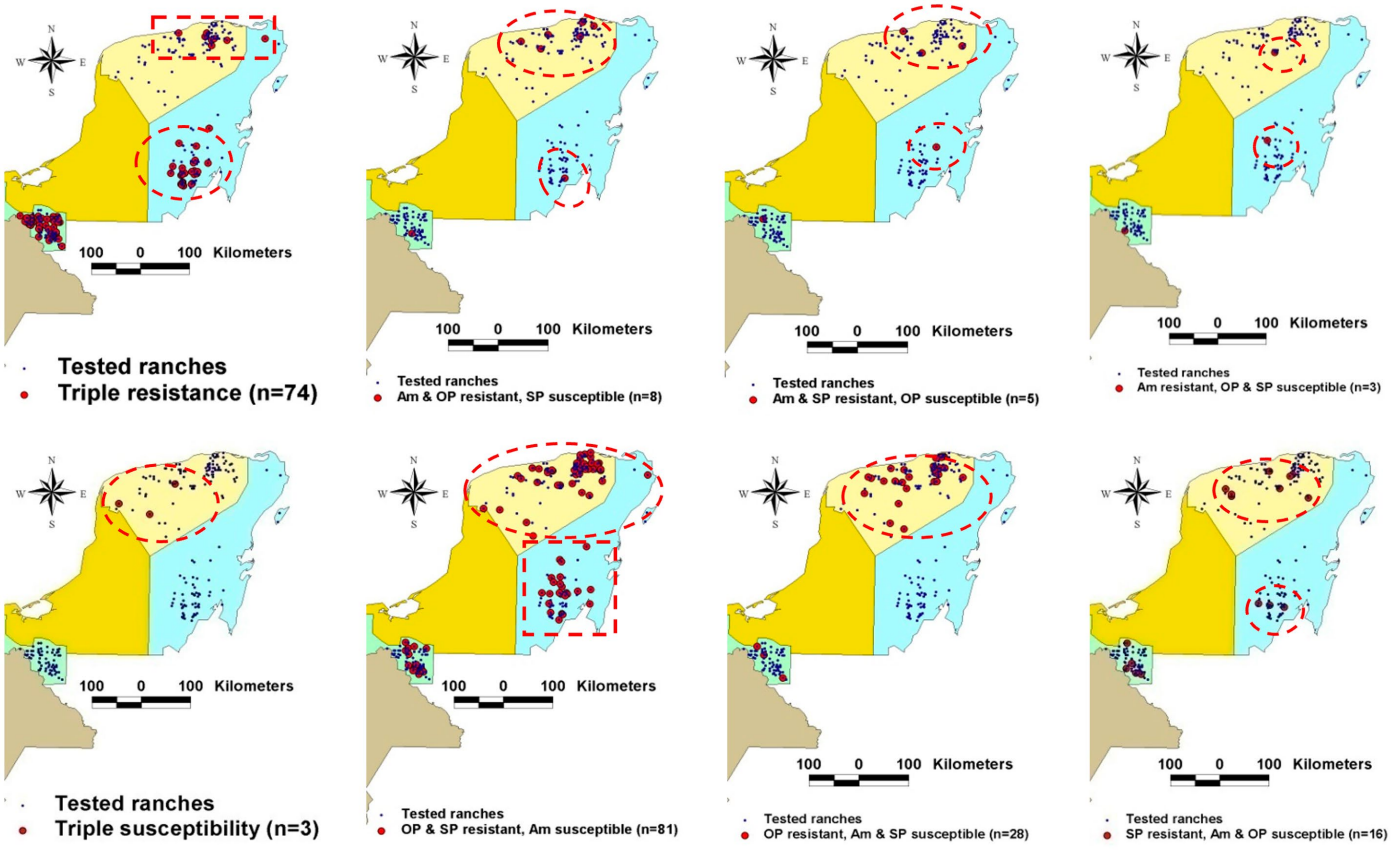
Disease dissemination is neither static nor homogeneously distributed over space. Data collected in Mexican farms where resistance to parasiticides used in bovines were geo-analyzed demonstrate heterogeneous geographical distribution even within the same region. Source: reference (22).

Because geo-referenced variables interact with one another, they help re-evaluate earlier theories. One example is disease prevalence, which is now shown to be neither geographically homogeneous nor static (22). For example, expressed as the prevalence of resistance against parasiticides, major differences are observed within the same region in the number of units (farms) that present simple, double or triple resistance (Figure 3).

Similarly, the intra-farm prevalence of bovine *Mycobacterium paratuberculosis* may differ up to 80% across farms—a finding associated with infective (epidemic) links (23). This means that measuring disease prevalence may be non-informative unless a specific (geo-referenced) region is identified within a specific timeframe.

### Section II. Applications: toward informative and cost–benefit related decision-making

#### Error prevention and extraction of new information

Aggregate data may induce errors. Because such data do not convey relationships, non-aggregate, point-based, high-resolution data are needed to investigate epidemics (24, 25).

The anticipatory creation of geo-referenced datasets that include relationships can facilitate cost-effective interventions (26, 27). For example, such datasets may include information of farm density, animal density, and road networks. In epidemics, analyses of such data can capture a much higher number of expected cases than alternatives (16).

**Figure 4:**
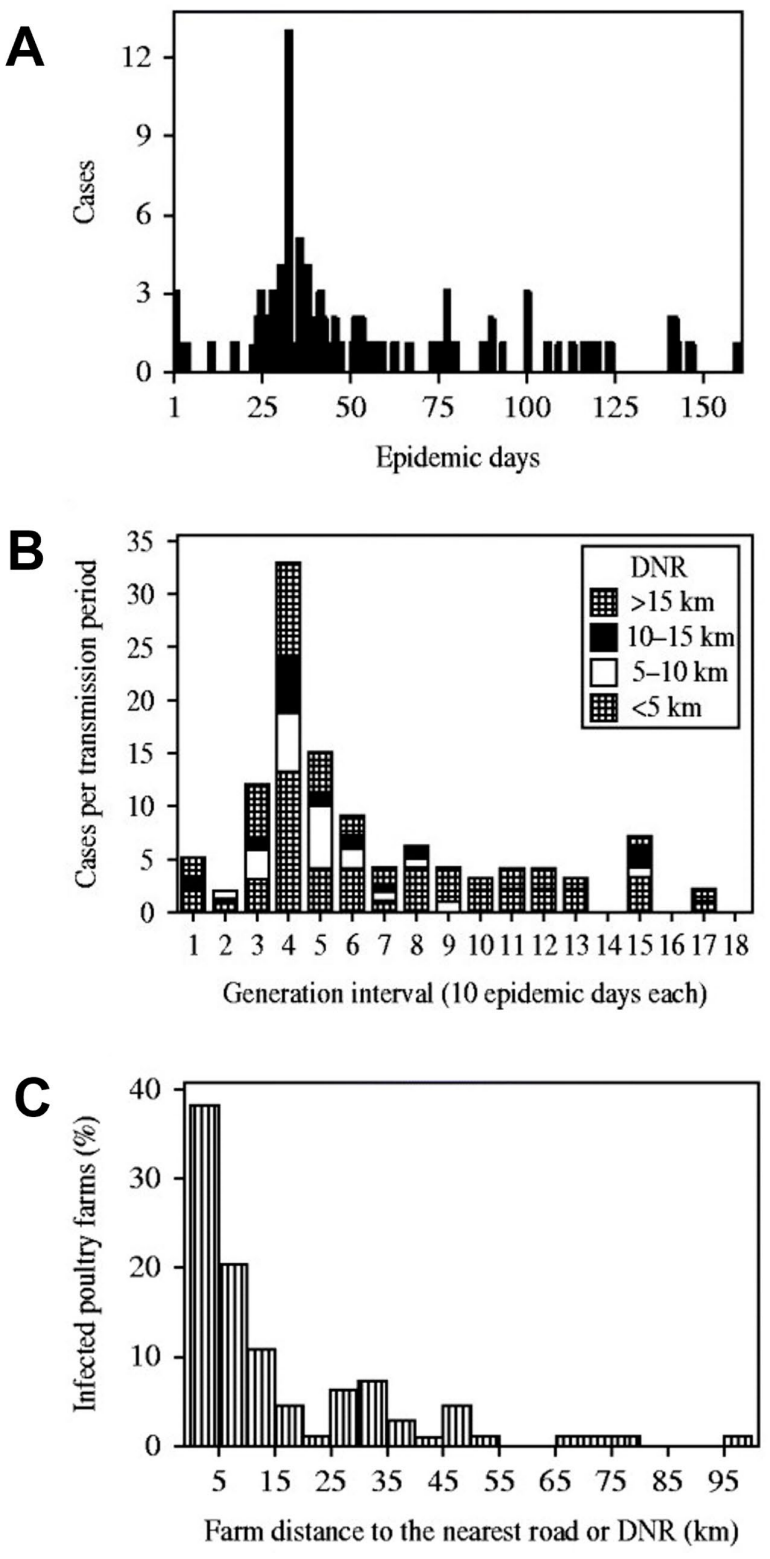
Error prevention and extraction of novel information. The Avian Influenza (H5N1) epidemic reported in Nigeria, in 2006, is shown with three visualizations. Plot **A** reports the classic approach, in which the number of cases is reported over time (where ‘epidemic day’ 1 is the first day a ‘case’ [an infected poultry far] was detected). Discontinuity is noticed in the first 70 epidemic days: in several days *no new case is reported*. Plot **B** reports the same data, now structured as ‘cases per transmission period’ (where each ‘generation interval’ is equal to 10 epidemic days). Now *cases are reported, without interruption, in the first 13 generation intervals (130 epidemic days)*. Plot **C** reveals new information: it demonstrates the relevance of proximity to a pre-established connecting structure: cases located at ≤5 km from the nearest road [DNR] represented 38% of all cases. Source: reference (17).

Because they can capture more dimensions than classic approaches, *bio-geo-dynamic assessments* are likely to *prevent errors.* For example, apparent gaps in the data (which suggested no new cases occurred several times in the first 70 epidemic days) seemed to occur when time was measured with chronological units (days, Figure 4A). Such patterns were not detected when the same data were reported as *generation intervals* (Figure 4B). When, instead of reporting hours or days, time was measured together with biological concepts (e.g., when the *transmission cycle* of the pathogen was considered), the previous gaps were no longer observed (Figure 4B). In addition, the distance between a specific case and the nearest connecting structure can also be captured (17). This geo-bio-temporal metric shows that the number of epidemic cases–expressed as proportion of all cases–, was inversely related with the distance between cases and the nearest road (Figure 4C).

#### Differentiation of infection types

Bio-geo-temporal analyses can also differentiate *infections*. At least five infecting types can be distinguished, which may prompt different interventions.

**Figure 5:**
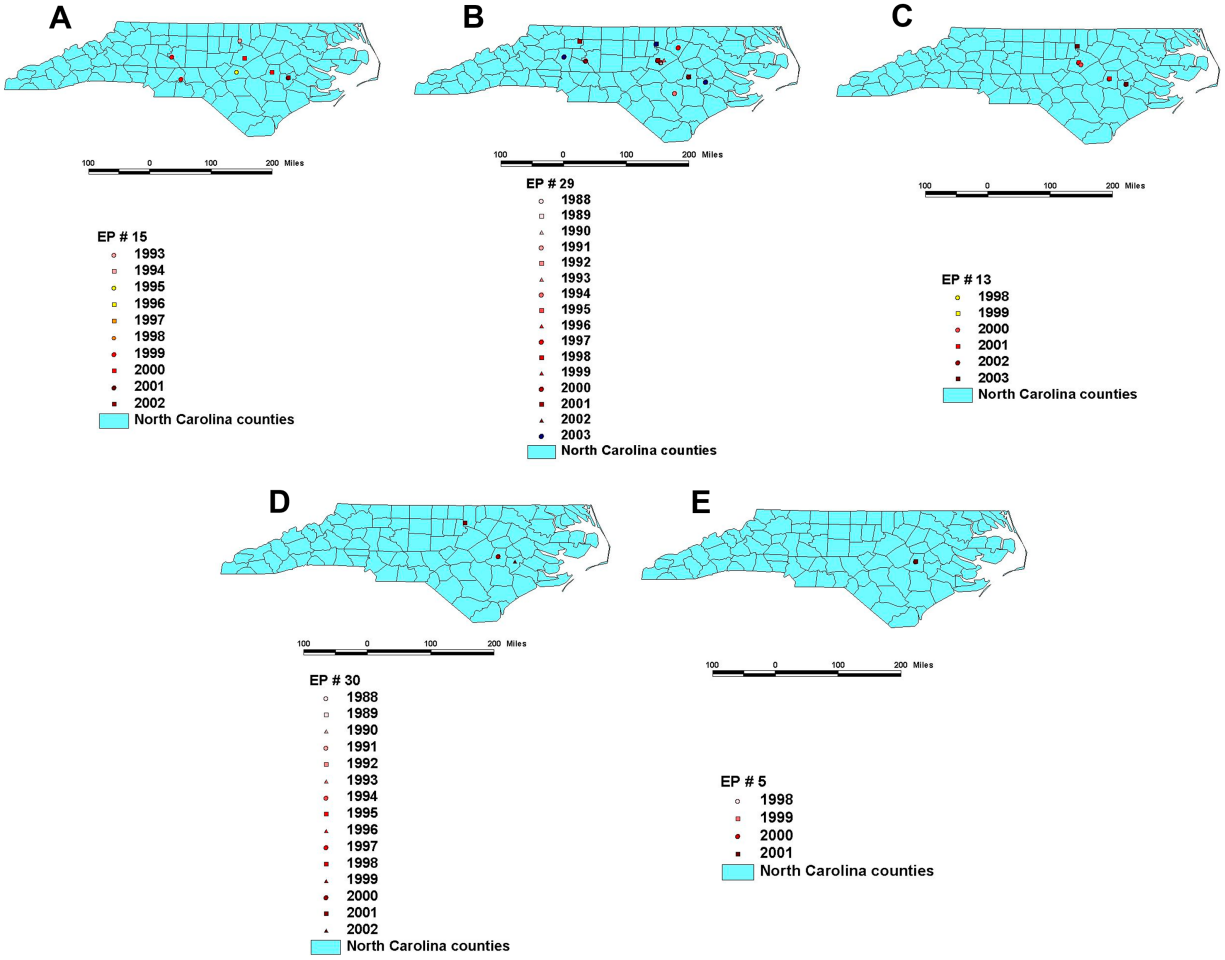
Temporal and geo-referenced data collected from farms located in North Carolina, United States support at least five classifications on infection types –four classified as non-spatial (strain-related), and one classified as spatial (local)–, which could facilitate geo-referenced, infection-type based decisions. Panels describe high (large) spatial and high (faster) temporal diffusion **(A)**, high spatial and low (slower) temporal diffusion **(B)**, low (small) spatial and low temporal diffusion **(C)**, low spatial and low temporal diffusion **(D)**, and local (not spatial) and frequent diffusion **(E)**. Maps display only the most recent observation on a given farm (previous observations on the same location may have occurred). Source: reference (29).

**Figure 6:**
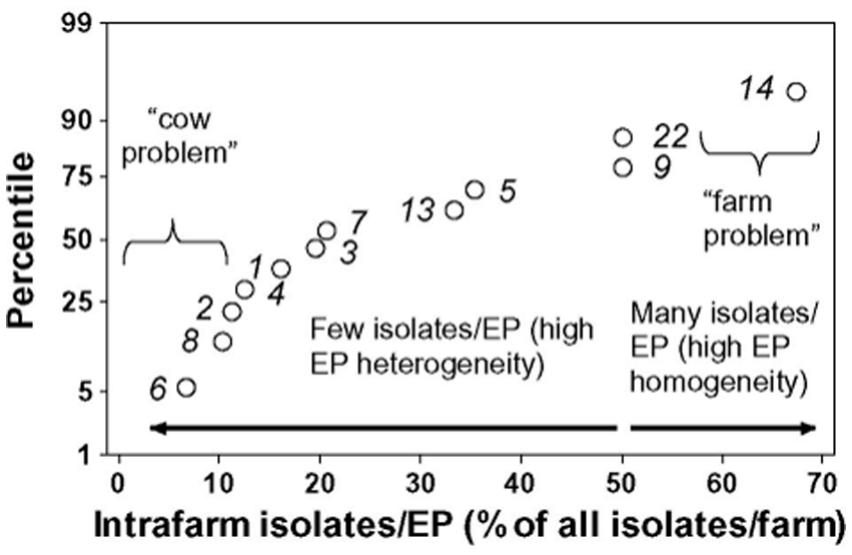
Sub-classification of infections within the local variety. The use of additional metrics (also generated by geo-referenced data) can further divide infections classified within the local variety. Source: reference (28).

For example, the detection of highly disseminating bacterial strains may lead to earlier, bacterial strain-specific interventions (Figure 5). Furthermore, two sub-varieties can be distinguished within the ‘local’ (no geographical spread) bacterial strain type. Based on the Heterogeneity Index (percent of intra-farm isolates that belong to the same bacterial strain), two subtypes can be differentiated: (a) ‘cow problem’ and (b) the ‘farm problem’ subtypes. A ‘farm problem’ is suspected when most bacterial strains found in a farm belong to the same strain but have not been found elsewhere (e.g., the percentage of isolates that belong to the same strain is higher than 50%, Figure 6). When the percentage of isolates that belong to the same strain is lower than 50% (when a large diversity of bacterial strains is found in the same farm, but they do not show spatial dispersal), a ‘cow problem’ is suspected (Figure 6).

If these analyses were frequently conducted, they could facilitate earlier (cost-effective) decisions. In one investigated case, decisions could have been made 5 years earlier, which could have prevented between *6 and 14 percentage points of disease occurrence* (28).

#### More effective and/or less costly decisions: continuity vs. contiguity

**Figure 7:**
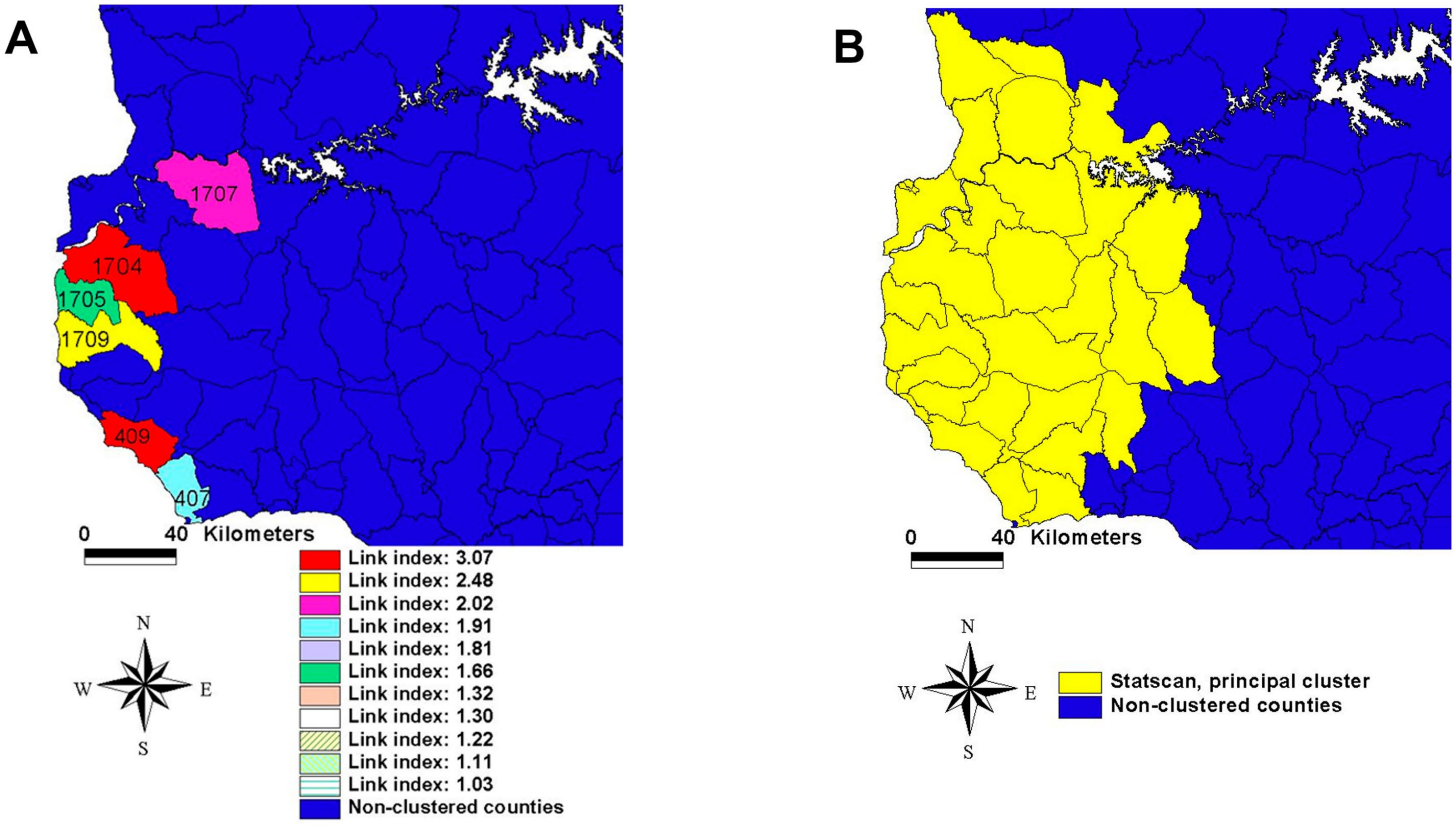
Continuity and contiguity in decision-making –the case of the 2001 Uruguayan FMD epidemic. Panel **A** displays counties that share both a continuous highway structure and link indices greater than 1.91. Panel **B** identifies a contiguous area identified statistically, in which the road network is not considered. These approaches result in a twice higher number of cases per km2 when connectivity is considered (left panel, with 0.0955 cases/km) than when only contiguity is estimated (right panel, 0.0456 cases/km^2^). Therefore, a 209.4% more effective control policy can be developed when aggregate data are avoided and, instead, connectivity is measured. Source: references (30, 31).

Bio-geo-temporal inferences based on continuous relationships can improve the validity and benefits of decision-making (30, 31). For example, twice as many cases can be detected per unit of area when connectivity is considered (Figure 7A) than when the local connectivity is ignored (Figure 7B).

#### More effective and/or less costly decisions: the ‘sandwich’ approach

**Figure 8:**
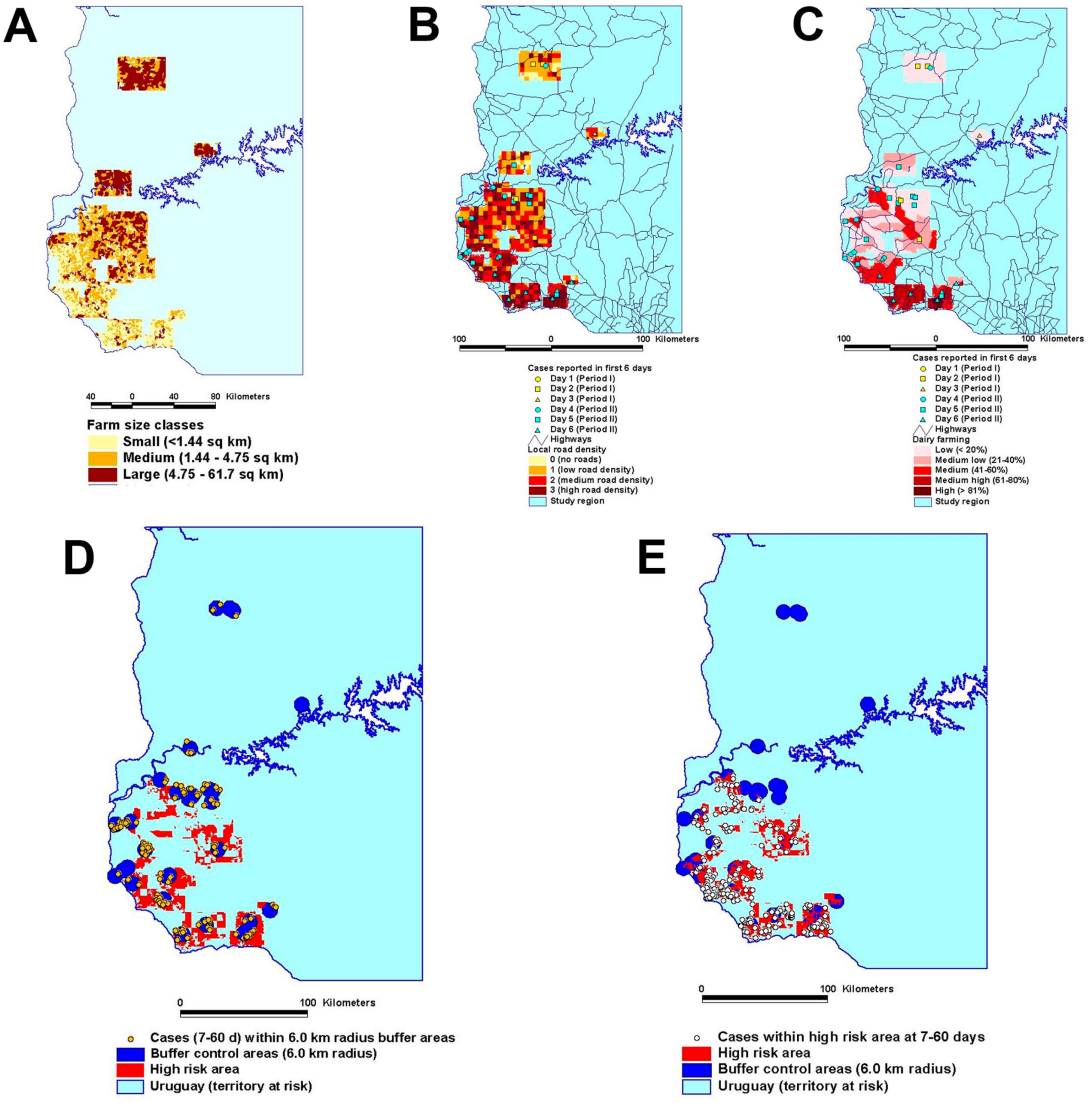
A composite (multi-dimensional) high-risk map was created, which considered farm size classes (A), the local road density reported in the first 6 epizootic days (B), and the local percentage of dairy farms (C). The number of cases reported at 7-60 epizootic days was then analyzed under two approaches that measured the same total area: (a) one that that was centered on the location of each earlier case (D) and (b) one that was based on the multi-dimensional approach (E). While focusing on the same total area, the multi-dimensional approach captured 1.77 times more cases than the non-multi-dimensional approach (198 vs. 112 cases, respectively). Source: (32).

Bio-geo-temporal analysis can detect multi-dimensional, complex relationships. For example, when the number of Foot-and-Mouth Disease (FMD) cases was classified according to four descriptors (farm size, animal density, county-specific percentage of dairy farms, and county road density [length of roads/county area]), a higher proportion of FMD cases were reported in areas characterized by (i) small and medium size land parcels, (ii) higher animal density, (iii) >20% farms specialized in dairy production, and (iv) high road density (Figure 8). By intersecting and linking together these classes, a higher proportion of cases can be found within a smaller proportion of the area to be controlled (32).

#### More effective and/or less costly decisions: enhanced detection of secondary cases

**Figure 9:**
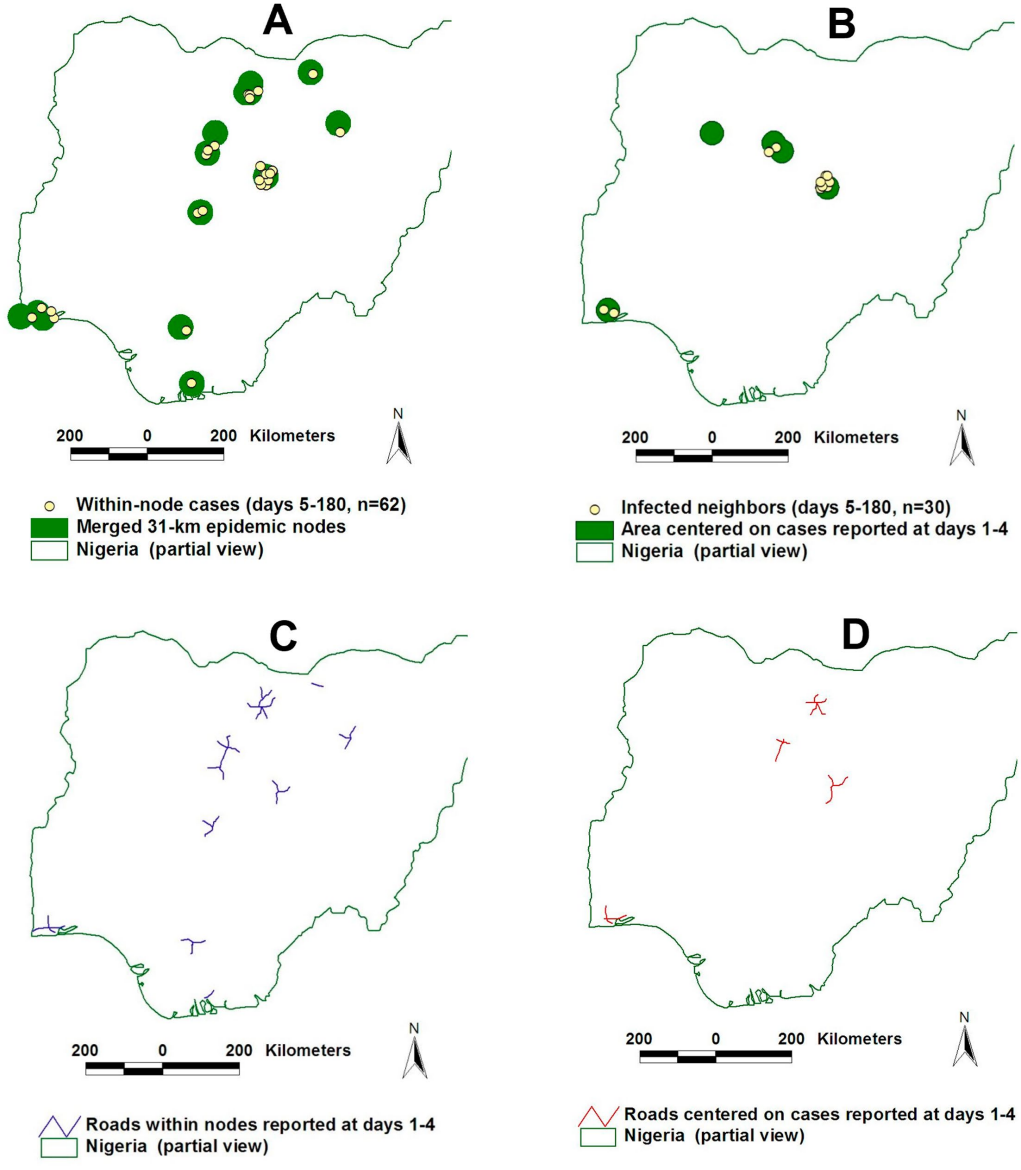
Differentiating connectivity from contacts –differences in the number of secondary and later cases detected and the length and fragmentation. After transmission cycle II, the connectivity model captured twice more cases of Avian Influence in Nigeria (62/30 or 206.6%) than the contact model **(A,B)**. The length of road segments was *three times longer* and less fragmented in the connectivity than in the contact model **(C,D)**. Source: reference (16).

While classic approaches emphasize only one or a couple of disciplinary perspectives, geo-epidemiology integrates all disciplines relevant to the study of disease dispersal and offers a visually explicit validation. For example, the hypothesis that *all cases have equal influence on disease dispersal* can be tested against the hypothesis that *highly linked epidemic nodes have more influence on disease dispersal than poorly linked nodes.* When tested with two procedures that create circles of identical area (one grounded on Network Theory, the other based on ‘near neighbor’ contacts), the Network alternative captured a *much longer* and *less fragmented* connecting structure than the contact alternative (Figures 9A–D).

#### Least costly, more effective detection of clusters of any geometric shape

‘Disease clusters’ have been defined as ‘hot spots’ that escape clear statistical or geometric definitions (33). Assumptions associated with ‘disease clusters’ include: (i) the view that disease dispersal is equally influenced by every primary case; (ii) future secondary cases (susceptible individuals) are always close to primary cases, so circles centered on the location of primary cases should capture secondary cases; and (iii) control circles of the same radius can apply to any epidemic, regardless of the infecting pathogen, affected species, geographical location and/or season. Following these assumptions, control circles of 3-km radius, centered on the location of an infected farm (‘primary’ case), have been imposed in European pig, poultry and bovine farms affected by different pathogens, at different times (34–36).

**Figure 10:**
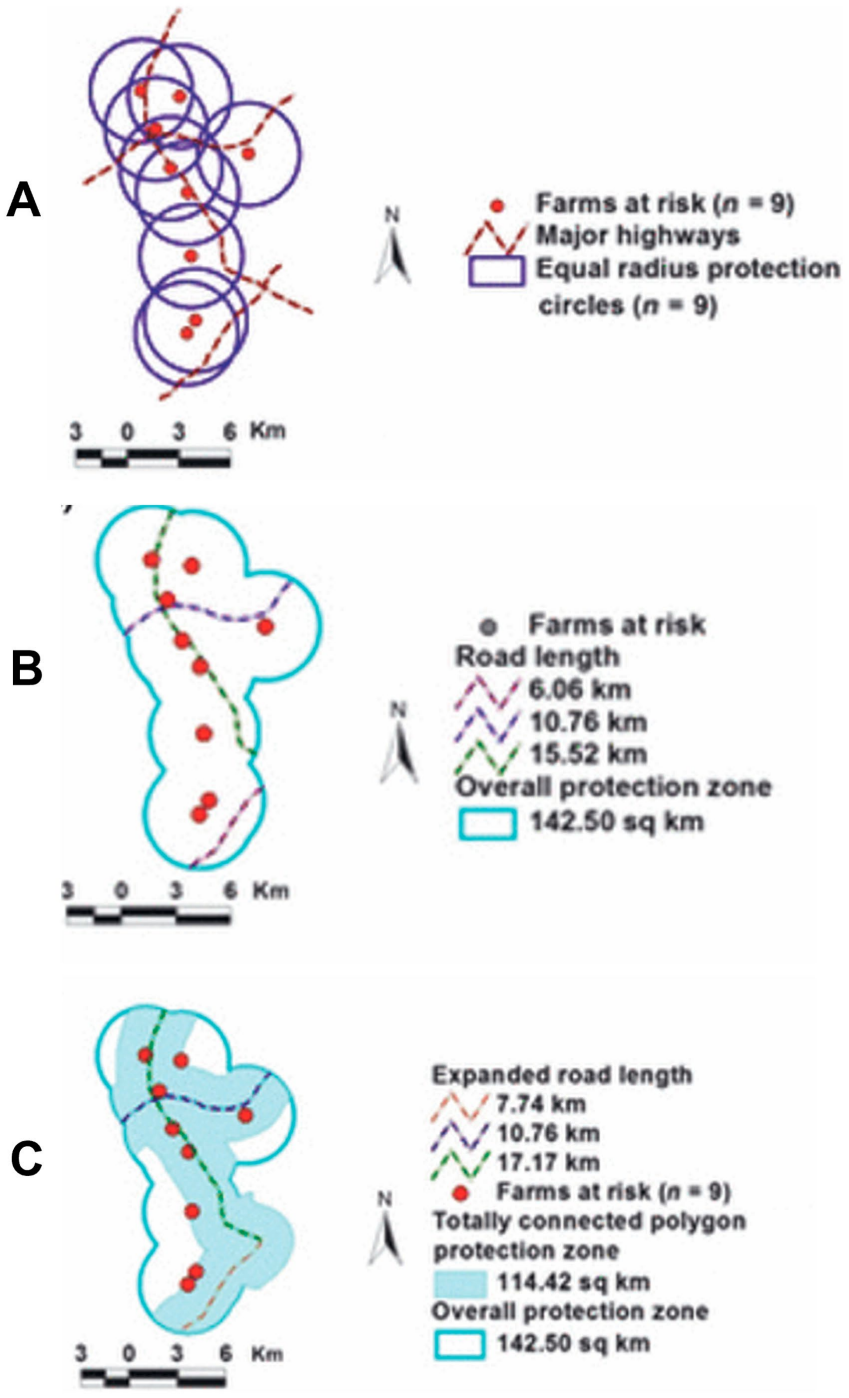
Benefit /cost analysis of control measures implemented in non-circular disease clusters. Circles of equal-radius (ER) were centered on the location of each of 9 English farms at risk of becoming infected (by Foot-and-Mouth Disease **A**). The location of major highways was also considered **(A)**. The length and degree of continuity of the road network as well as the overall protection zone (which partially included overlapping circles) were estimated (142.5 km2, **B**). Because the connecting road structure was interrupted in the south-eastern corner of the control area, an alternative solution expanded such area by creating a new circle centered on the road segment required to achieve complete (non-interrupted) road continuity **(C)**. The new strategy (centered on roads, not farms at risk) resulted in a 20% smaller (114.42/142.5 km2 or 0.8) overall area to be controlled (‘cost’), which displayed a 123.6% density of farms at risk/km2 (0.078 farms per sq. km/ 0.0631 farms per sq. km or ‘benefit’) or 54.5% greater benefit/cost ratio (123.6/0.80). Source: reference (36).

Yet, such policies can miss non-circular disease clusters (37). In contrast, Figure 10 shows that bio-geo-temporal assessments can estimate the benefit/cost ratio of interventions applied to geometrically irregular disease clusters, even in very small and non-circular infected areas (38).

#### Rapid design and implementation of emergency vaccinations with limited resources

Cost–benefit oriented approaches are especially required when resources are limited and urgencies emerge, such as unexpected vaccinations. One such a situation was experienced in Tanzania, in 2018, when an outbreak of human rabies started close to a major urban center (38).

**Figure 11:**
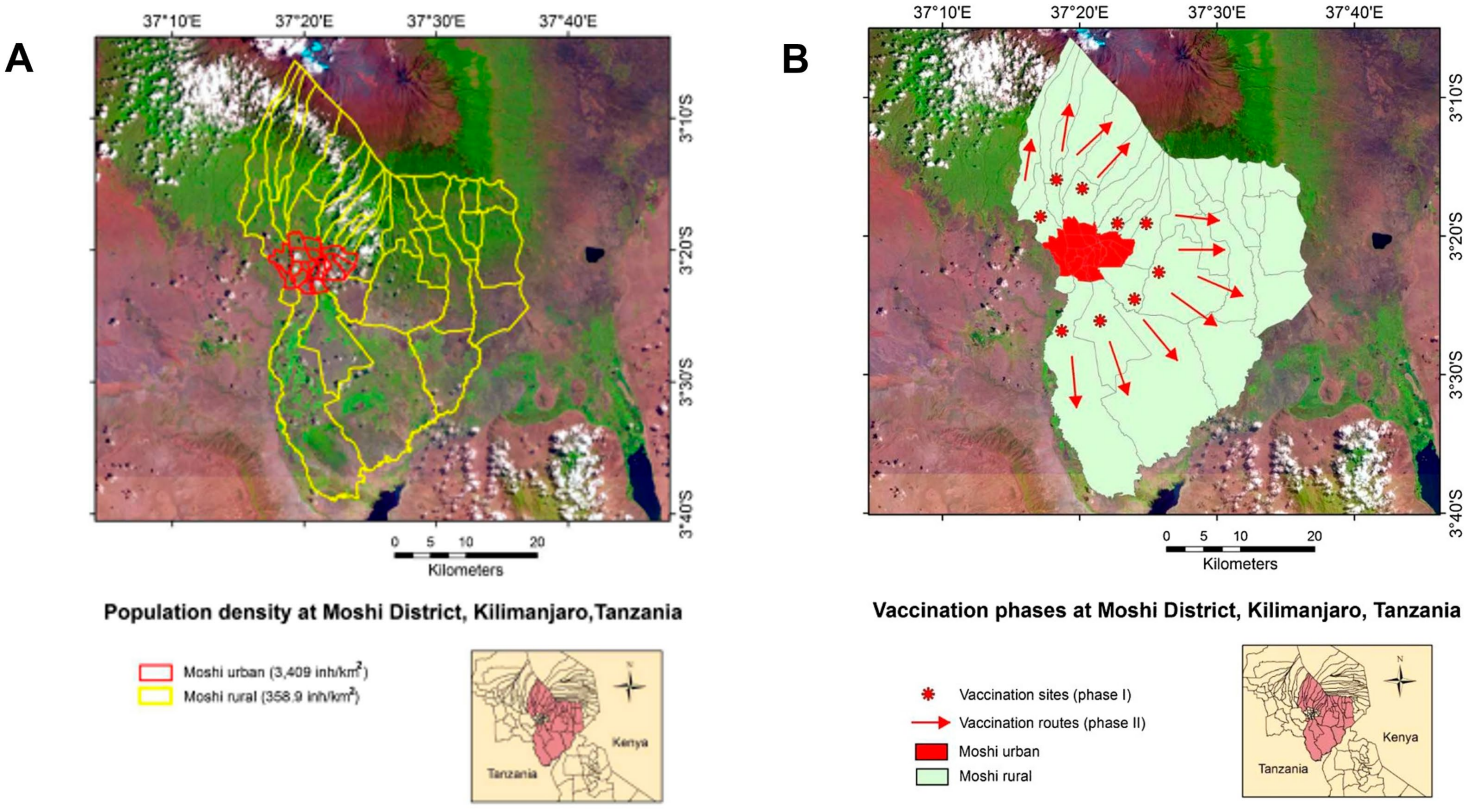
Emergency anti-rabies vaccination in northern Tanzania. The north-eastern region of Tanzania is shown, including a satellite photo of Mt. Kilimanjaro, on the northern edge, and a red contour that identifies the city of Moshi **(A)**. The rabies outbreak was controlled within the rural area of the Moshi district, identified by a yellow contour **(A)**. A two-phase campaign was implemented: after vaccinating along a ‘ring’ outside Moshi (circles, **B**), additional vaccinations were conducted in the countryside (arrows, **B**). Contrary to classic and static approaches, this campaign did not start where population density was the highest but along a ‘ring’ where population density was much lower. Consequently, this strategy could be completed faster and a lower cost than a vaccination implemented in a place with 10 times more population. Source: reference (38).

Then, the adopted strategy first implemented a ‘ring’ vaccination near to but outside a major urban center, which later expanded into the low-density, rural area comprised between the ‘ring’ and Mount Kilimanjaro (Figure 11). By containing the virus within two ‘walls’ (the ring vaccination on one side, the Kilimanjaro on the other), the time involved in implementing this strategy was negligible compared to standard practices. No rabies-related case was reported in the vaccinated area for over a year and the cost of the 2018 Tanzanian campaign was 3.28 times lower than anti-rabies vaccinations implemented in similar environments (38).

#### Applications in human diseases: test positivity-based, cost-effective interventions

Geo-epidemiology may also apply to human medicine. For example, Chinese geo-referenced and temporal data on COVID-19 have revealed Network properties (19).

Geo-epidemiology may also be instrumental in solving a major problem encountered in many epidemics. That is when many of the infected individuals are asymptomatic. As seen in COVID-19, asymptomatic individuals are major disease disseminators: they are not aware that they are infected and do not request medical assistance (39). Such a situation creates a deceiving consequence: diagnostic tests tend to be conducted among symptomatic, not among asymptomatic individuals.

This situation induces high percentages of test positivity (TP or percentage of tested individuals that yield ‘positive’ tests), even when ‘positive’ individuals are less likely to disseminate the disease than asymptomatic ones. Consequently, high TP percentages do not necessarily reflect the true status of the population but the status of those that seek testing.

The alternative to testing 100% of the population on a given day—usually, an unfeasible goal—is to test as much as possible, so low percentages of the TP are found in many areas and only one (or very few) area(s) display high TP percentages. When such a situation is found, the prompt removal (isolation) of positive cases located in the central area may prevent disease dissemination into neighboring areas. Geo-epidemiology could implement such a strategy (39).

**Figure 12:**
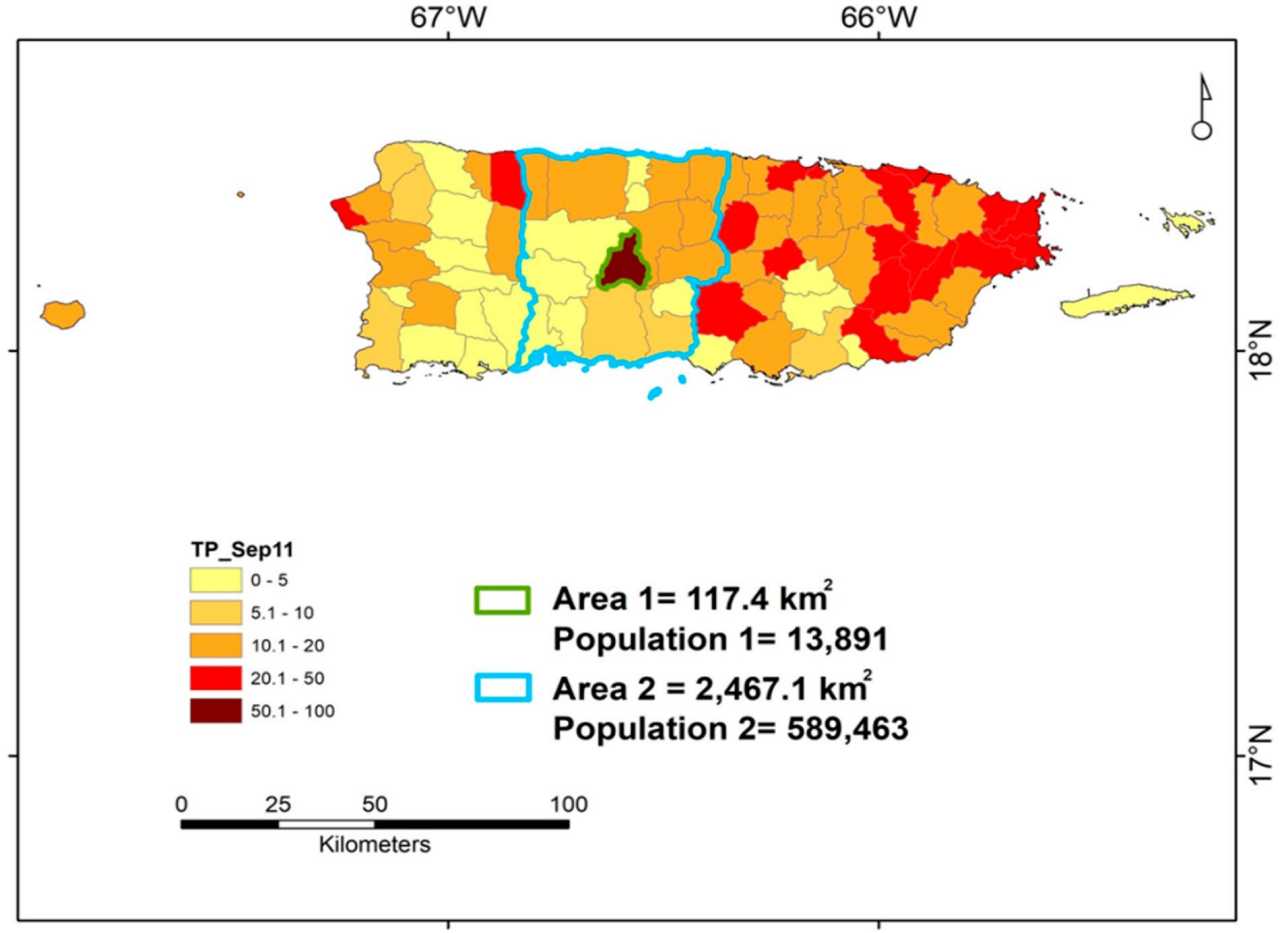
Geo-referenced, cost–benefit oriented assessment of Test Positivity—an example based on data collected in Puerto Rico. The county-level data on Test Positivity (percentage of positive cases over all individuals tested) reported in Puerto Rico on September 11, 2020 is shown. Area 1 identifies the municipality of Jayuya. Area 2 identifies the surrounding region, which includes ~42 times more people and covers an area ~ 8.5 times larger than those of Jayuya. Given Jayuya +50% TP, a greater testing effort conducted in this municipality may yield large benefits, which may also include the surrounding area (Area 2) and, indirectly, benefit the western half of the island. Vice versa, keeping the same level of testing performed before may lead to long-lasting, costly consequences: if the virus circulating within Jayuya reached the surrounding area and the prevalence of COVID-19 became similar to the one affecting Jayuya (which is estimated by test positivity), then a much larger number of people (up to 42 times larger) could become infected, who would reside in an area 8.5 times larger, i.e., control would then be much harder, longer and costlier. Source: reference (39).

To reduce disease dispersal at the lowest cost and/or in the shortest period, a double approach could consider (i) county-level, temporal and geo-referenced data on test positivity.

(TP), and (ii) cost–benefit related considerations (39). This strategy could focus not on spending resources equally and constantly across all areas but, instead, it could briefly concentrate resources in a small area where the TP is substantially higher than the surrounding area (Figure 12).

#### Applications in zoonoses

**Figure 13:**
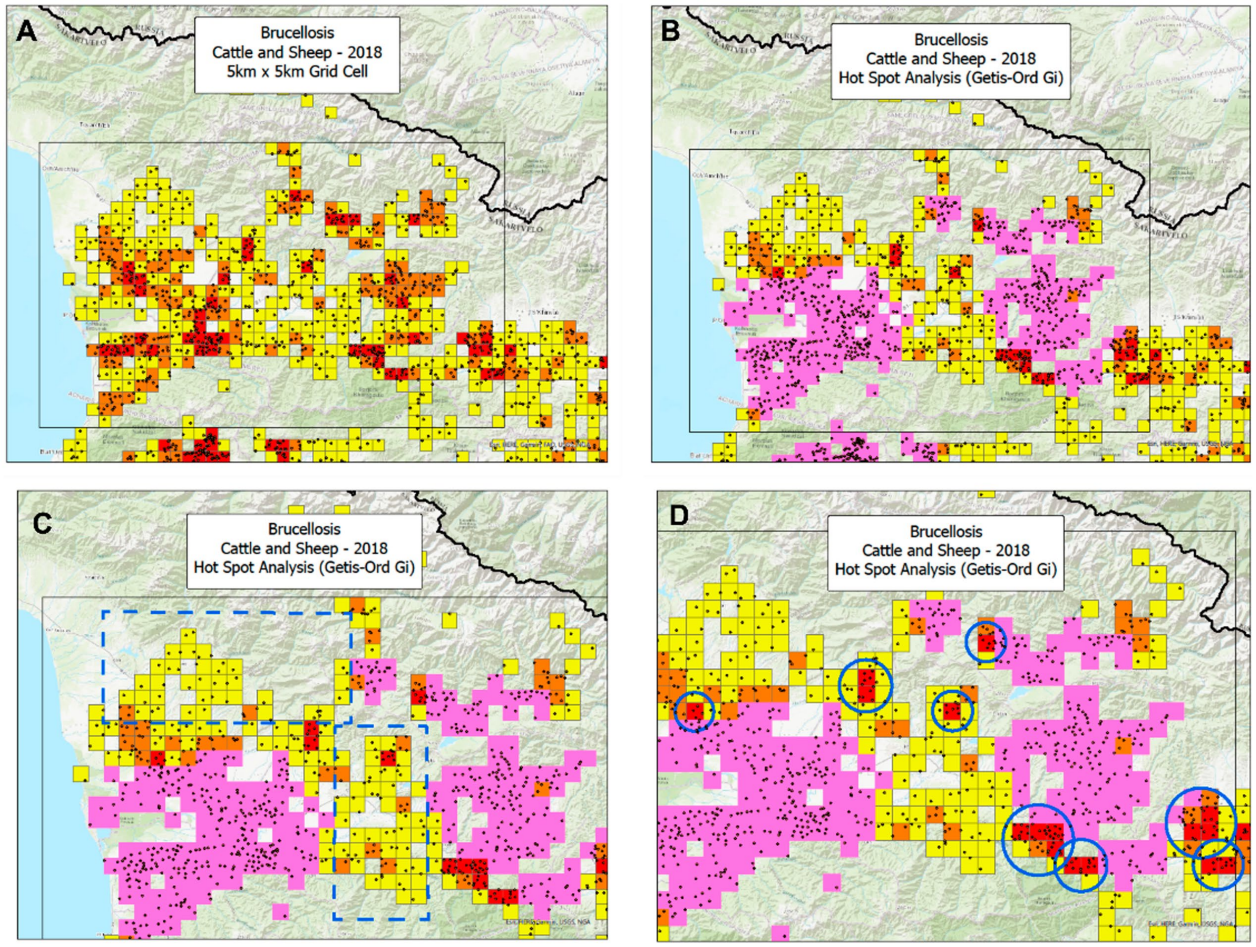
Temporal-bio-geographical based decision-making in zoonoses. Based on data on brucellosis collected in the Republic of Georgia, two methods were compared: the same data were analyzed by the bio-geographical method (BG) and the Hot Spot analysis (HS). Ruminant data from 2018 show that the BG method identified 139 25-square kilometer orange and red squares, which included 583 cases or 4.19 cases/square (583/139, **A**). In contrast, the HS analysis found 521 cases in 194 squares or 2.68 cases/square (521/194, **B**). Consequently, the case density of the BG approach was 56.3% higher (4.19/2 than that of the HS). This difference in potential cost-effectiveness was explained by two factors: (i) the HS missed large areas that included numerous cases **(C)** and, (ii) in particular, the HS analysis missed nine mini-areas with a very high case density **(D)**. Such a difference was achieved while the BG analysis occupied an area 28.4% smaller (139/194 squares or 71.6%) than the area covered by the HS analysis. If cost effectiveness of interventions was measured as the ratio of benefits over costs (here expressed as cases captured/area unit), then the ratio of the BG method would be 2.18 (156.3/71.6), i.e., the BG method exhibited a benefit/cost ratio twice as large as the one shown by the HS analysis. Zoonotic sites (geographical locations where human and non-human cases were detected) displayed a case density between 2.6 and 2.99 times as high as any site that only included non-zoonotic cases (i.e., those that only reported human, cattle, or sheep cases, **E**). Source: reference (40).

Geo-referenced, cost-effective decisions may prevent zoonoses (40). They can detect more cases in smaller areas than alternatives (Figures 13A–D).

**Figure 14:**
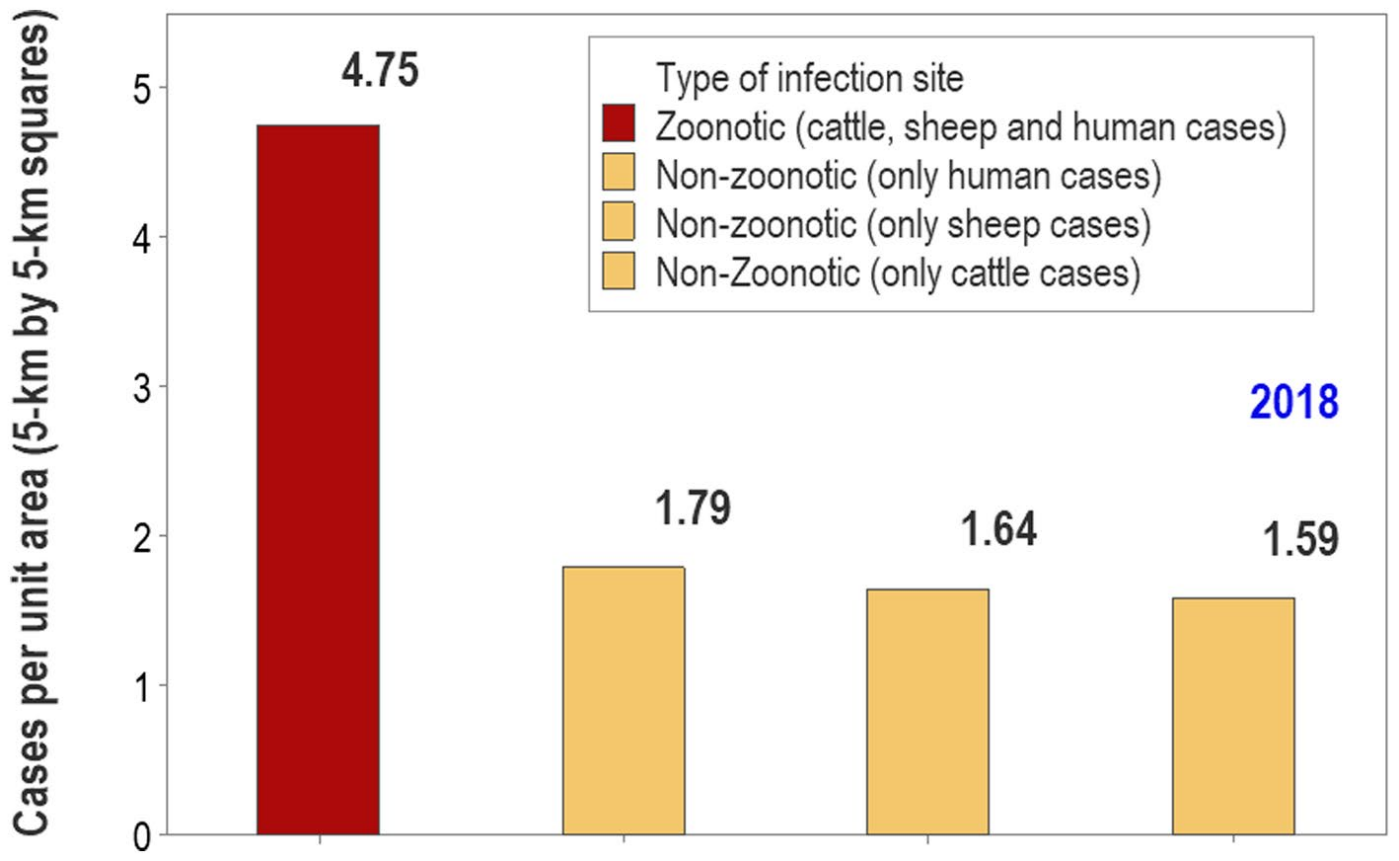
A bio-geographical analysis may guide cost–benefit oriented, prioritized interventions. Using a bio-geographical method that explores the contribution of site types to the data, it is shown that, in 2018, brucellosis-related zoonotic sites (geographical locations where human and non-human cases were detected; Figure 13) displayed a case density between 2.6 and 2.99 times as high as any site that only included non-zoonotic cases (i.e., those that only reported human, cattle, or sheep cases). This metric may inform decision-makers how, where and when interventions may be prioritized. In this scenario, sites that report a high density of zoonotic cases could be the first priority, followed by sites where non-zoonotic cases are reported with a high case density, and finally, sites that report a low case density involving any species. Source: reference (40).

Analyses that integrate bio-geo-temporal data could identify *where* and *when* to intervene at *lower cost*/*greater benefit*. As shown in Figure 14, when geographical locations that report human and non-human brucellosis cases are considered, zoonotic sites may exhibit a much higher case density and, therefore, should be prioritized in interventions (40, 41).

### Section III. Teaching that supports inter/trans-disciplinary research and vice versa

#### Balancing cost with effectiveness

To ensure feasibility, the implementation of proposed educational programs should also address existing systemic challenges, including resource availability, interdisciplinary collaboration barriers, and the integration of new curricula into established academic frameworks. Leveraging partnerships with organizations experienced in GIS education and public health could accelerate the development of such programs.

#### Method development: the DIKW (data, information, knowledge, ‘wisdom’) process

To be used, *data* should be transformed into *information*, later reformatted as *knowledge* and, finally, applied. The DIKW (*data, information*, *knowledge*, ‘*wisdom’*) process could move epidemiology from data-based into knowledge-rich inputs that inform decisions (42, 43).

To achieve it, new programs could consider learning-related aspects, such as: (i) pattern recognition, (ii) knowledge creation/interpretation/integration, and (iii) knowledge use (44, 45).

#### Visual language and interdisciplinary problem-solving

The aspects mentioned above may develop new interdisciplinary language (46). Because visualizations convey information interpretable across disciplines, geo-referenced, *visual* data may promote learning, research and problem-solving (47–50).

#### Georeferenced disease datasets that foster research and education

The anticipatory creation of disease-related, bio-geo-temporal datasets may also foster method development and critical thinking. Cognitive skills that foster data analysis are now taught even in secondary schools (51, 52). Educational and research programs on geo-epidemiology may emphasize problem-solving (53–55).

To optimize learning, the dynamic complexities associated with changing epidemic processes should be addressed in the language used in *educational practices* (56, 57). Because they inform on numerous and dynamic *relationships*, visually explicit teaching formats seem more appropriate than static alternatives (58). Bio-geographical teaching strategies promote inter-personal skills, critical thinking, and knowledge discovery (59, 60).

Building and teaching how to use geo-epidemiological tools is globally needed (61). Because many health-related graduate programs were created before COVID-19 emerged, adjusting learning environments to pandemic-related learning needs may be necessary (62, 63).

#### Is global graduate education on geo-epidemiology both needed and feasible?

COVID-19 was and still is a tragic lesson: it revealed major gaps in scientific knowledge (64). Bibliographic searches provide indirect but strong hints on probable omissions: when the keywords ‘geo-referenced’ and ‘COVID’ were searched for, on August 27 of 2024, the *Web of Science* only retrieved 29 hits. They represented 0.00005% of all the literature on COVID-19 published at that time (29 / 524,284). One likely explanation for such a cognitive gap is the lack of educational programs on geo-epidemiology.

The need for visually explicit, *data-driven education* on disease dispersal has been reported (65). While traditional teaching cannot be *scaled up*, online education can (66). Data- driven, online, student-centered education may promote critical thinking as well as validation and lifelong, question-generating skills (67).

New educational programs on geo-epidemiology may be rapidly developed because *five conditions or resources* are already mature and available: (i) a large, inter-disciplinary group of educators/researchers, (ii) international libraries on disease-related datasets, (iii) a methodology that integrates theory with operations applicable to many diseases affecting human and non-human species, (iv) many research publications that offer numerous examples of cost–benefit oriented interventions, and (v) the ability to develop and use context-specific software.

Based on electronic platforms, new educational programs can be offered at low or negligible costs. Using such formats, geo-epidemiology could provide new interdisciplinary programs, which also capture *One Health* dimensions (68, 69).

## Limitations

Numerous tools and research findings likely to influence geo-epidemiology have not been comprehensively examined here. They include: (i) new sources of geo-referenced disease data (70, 71) and (ii) new algorithms that address combinatorial problems (72–74).

## Summary and conclusion

The theoretical foundation, operational consequences, and educational needs associated with geo-epidemiology are summarized. At least two emphases characterize bio-geo-temporal assessments: (1) the analysis of connecting structures established before disease emergence, (2) measures that facilitate site-specific, cost/benefit-related decision-making. It is suggested that new, data-driven, participatory educational and research programs may foster earlier, less costly, and/or more effective interventions against disease dispersal.

## Data Availability

Publicly available datasets were analyzed in this study. This data can be found at: Links and citations to the papers that reported the original data are provided.
